# Characterization of the complete chloroplast genome of *Salix linearistipularis* (Franch.) Hao 1936

**DOI:** 10.1080/23802359.2021.1967809

**Published:** 2021-08-24

**Authors:** Rongrong Ren, Xiaoping Li

**Affiliations:** a Collaborative Innovation Center of Southern Modern Forestry, Nanjing Forestry University, Nanjing, China; b College of Forestry, Nanjing Forestry University, Nanjing, China; c Key Laboratory of Forest Tree Genetics and Breeding and High-Efficiency Cultivating in Jiangsu Province, Nanjing, China

**Keywords:** Chloroplast genome, genome sequencing, *Salix linearistipularis*, Salicaceae, phylogenetic analysis

## Abstract

We characterized the complete chloroplast genome of a perennial woody plant species, *Salix linearistipularis*, based on high-throughput sequencing and *de novo* assembly technology for the first time. The complete chloroplast genome of *S. linearistipularis* is 155,564 bp in length, comprising one large single-copy region (LSC, 84,460 bp), one small single-copy region (SSC, 16,182 bp), and two inverted repeat regions (IRA and IRB, 27,461 bp). The GC content of the whole chloroplast genome was 36.69%. This chloroplast genome encodes a total of 132 genes, including 86 protein-coding genes, eight ribosomal RNA genes, and 37 tRNA genes. Phylogenetic analysis reveals that *S. linearistipularis* is grouped with 13 other *Salix* species in Salicaceae.

*Salix linearistipularis* (syn. *S. mongolica*) is a shrub or small tree classified in the genus *Salix* of the Salicaceae family that is found in Inner Mongolia, Mongolia, three northeastern provinces, and (Far East) Russia. As the perennial woody species naturally distributed in the saline-alkali soil of the Songnen Plain in Northeast China (Ishida et al. 2009), *S. linearistipularis* exhibits strong endurance to salt stress and easy reproduction. Thus, it is broadly used for landscaping, alkali soil improvement, sand fixation, and reforestation with high ecological and economic benefits (Nan et al. 2016). Furthermore, the identification of genes for stress tolerance in this dioecious plant can facilitate the study of sex differentiation (a.Feng et al. 2020) and sex-related salt tolerance mechanisms (b.Feng et al. 2020). Nevertheless, due to the highly efficient crossing rate among *Salix* species, the classification of the genus *Salix* spp. is still disordered (Chen et al. 2010). Fortunately, the chloroplast genome of plants is conserved across evolution and has been widely and reliably used to evaluate relationships between closely related species. In this study, the complete chloroplast genome of *S. linearistipularis* was characterized for the first time to further investigate the genetic background of this species. The data will set a molecular foundation for the exploitation and conservation of willow resources.

Fresh leaves of *S. linearistipularis* were sampled from Yanchi County, Ningxia, China (37°47'N, 107°25'E) for DNA extraction. The extracted DNA was stored at −80 °C at the Key Laboratory of Forest Genetics and Biotechnology at Nanjing Forestry University. The voucher specimens were deposited in the herbarium of Nanjing Forestry University.

(https://www.njfu.edu.cn/, voucher number: NXHL2017003; Xiaoping Li, xpli@njfu.edu.cn). Whole genomic DNA was extracted from fresh leaves of *S. linearistipularis* by a modified CTAB method (Doyle and Doyle 1987) and fragmented to construct a 2 × 150 bp library for Illumina HiSeq sequencing (Illumina, San Diego, CA). After filtering the 5,574,054 raw reads, 4,848,768 high-quality reads were assembled by NOVOPlasty software

(https://github.com/ndierckx/NOVOPlasty) (Dierckxsens et al. 2017). The filtered scaffolds were aligned, oriented, and combined based on overlapping regions to construct the complete circular chloroplast genome sequence. The assembled chloroplast genome sequences of *S. linearistipularis* were uploaded to the online software GeSeq (https://chlorobox.mpimp-golm.mpg.de/) (Tillich et al. 2017) for preliminary annotation, and the initial annotation results were manually modified by comparison with the chloroplast genome of *S. gordejevii* (MW562004). The complete chloroplast genome sequence was submitted to GenBank under the accession number MZ018223. To determine the phylogenetic position of *S. linearistipularis* in Salicaceae, the chloroplast genome sequences of *S. linearistipularis* and 32 members of the Salicaceae family were aligned by MAFFT v.7.475 (Katoh and Standley 2013) (https://mafft.cbrc.jp/alignment/software/). Then, phylogenetic analysis based on the maximum-likelihood (ML) method was executed in MEGA X (Kumar et al. 2018) with 1000 bootstraps. *Itoa orientalis* in the family Flacourtiaceae served as the root.

The whole chloroplast genome of *S. linearistipularis* is 155,564 bp in length and exhibits a typical quadripartite structure comprising one large single-copy region (LSC, 84,460 bp), one small single-copy region (SSC, 16,182 bp), and two inverted repeat regions (IRA and IRB, 27,461 bp). The GC content of the whole plastid genome was 36.69%, and the respective contents of the LSC, SSC, and IR regions were 34.41%, 31.03%, and 41.86%. The chloroplast genome of *S. linearistipularis* encoded a total of 132 genes, including 86 protein-coding genes, eight rRNA genes, and 37 tRNA genes. The *ycf1* gene is located at the IRa/SSC border as a pseudogene. One *rps12* gene is split into two individual transcripts. While the majority of genes are present in single-copy form, four rRNA genes, seven tRNA genes, and eight protein-coding genes have two copies. A total of 18 genes contained introns, three of which (*rps12, ycf3, clpP*) included two introns, and the remaining 15 genes contained only one intron.

The results of phylogenetic analyses yielded further evidence of the phylogenetic taxonomy in Salicaceae. As illustrated in Figure 1, two major clades were identified with strong support values (BP = 100%). Clade I contained two subclades: Subclade I (BP = 100%) and Subclade II (BP = 96%). Within Clade II, 19 species in *Salix* were separated into two subclades (BP = 100%). The first subclade included *S. interior*, *S. tetrasperma*, *S. paraplesia* and *S. chaenomeloides*. Within the second subclade, *S. arbutifolia* was placed as the basal species and clustered with the other 14 species in *Salix* (BP = 100%). Here, *S. linearistipularis* was grouped with 13 other *Salix* species in Salicaceae *(S. suchowensis*, *S. argyracea*, *S. eriocephala*, *S. rehderiana*, *S. sinopurpurea*, *S. gracilistyla*, *S. brachista*, *S. minjiangensis*, *S. hypoleuca*, *S. integra*, *S. gordejevii*, *S. magnifica*, *S. rorida*) (BP = 73%).

**Figure 1.**
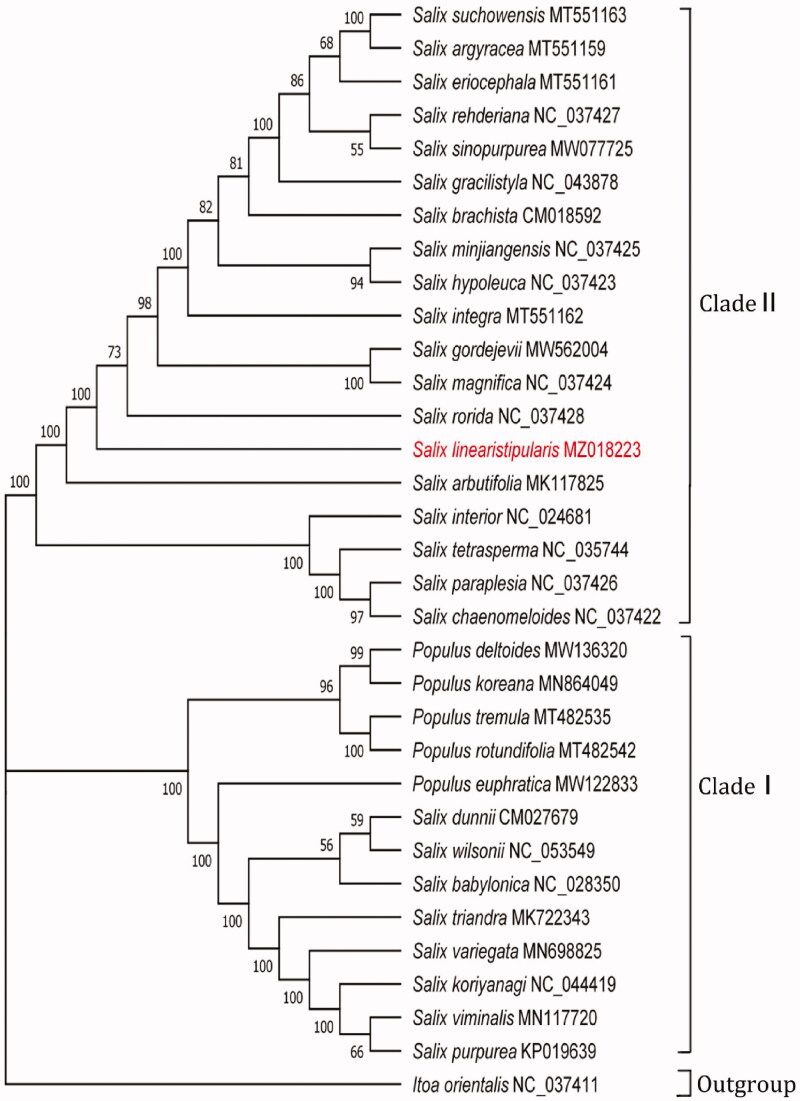
Maximum-likelihood (ML) phylogenetic analysis of 32 complete chloroplast sequences of Salicaceae species using *Itoa orientalis* as an outgroup with 1000 bootstraps.

## Geolocation information

Nanjing Forestry University, 159 Longpan Road, Xuanwu District, Nanjing City, Jiangsu Province

## Data Availability

The data that support the findings of this study are openly available in GenBank of NCBI at https://www.ncbi.nlm.nih.gov/nuccore/MZ018223.1/, reference number MZ018223. The raw sequence data used in this research were deposited successfully with registered numbers of associated BioProject, SRA, and Bio-Sample: PRJNA734150, SRR14700532, and SAMN19473694, respectively.
